# The I COPPE Scale Short Form for measuring multidimensional well‐being: Construct validity and reliability from US, Argentinian, and Italian large samples

**DOI:** 10.1002/jcop.22659

**Published:** 2021-07-05

**Authors:** Ciro Esposito, Immacolata Di Napoli, Salvatore Di Martino, Isaac Prilleltensky, Caterina Arcidiacono

**Affiliations:** ^1^ Department of Humanities University of Naples Federico II Naples Italy; ^2^ Division of Psychology University of Bradford Bradford UK; ^3^ Department of Educational and Psychological Studies University of Miami's School of Education & Human Development Miami Florida USA

**Keywords:** cross‐cultural comparison, multidimensional well‐being, short form measures, structural equation modeling, validity and reliability

## Abstract

The aim of this study is to present a short form of the I COPPE scale of multidimensional well‐being. We conducted two studies, which include four samples collected across three countries, namely United States, Argentina, and Italy. In the pilot study we tested during the data analysis phase whether it was feasible to reduce the full I COPPE scale by omitting the items dealing with past well‐being. Prompted by the positive results of the pilot study, we launched a final validation study with a sample of 2682 Italian people who completed the I COPPE scale short form, which is designed without items referring to past well‐being. Results from a series of confirmatory factor analyses show that the I COPPE scale short form presents acceptable levels of construct validity and reliability. Moreover, the 7‐factor correlated‐trait model proved to be the best fit for the data. We discuss advantaged of using the I COPPE scale short form along with limitations and future recommendations.

## 1 INTRODUCTION

In recent years, we have witnessed a shift from unitary models of subjective well‐being to well‐being as a multidimensional construct (Arcidiacono & Di Martino, 2016; Sarriera & Bedin, 2017). Ryff (1989) was amongst the first to propose psychological well‐being as an optimal psychological functioning based on six dimensions: self‐acceptance, positive relationships, autonomy, control over one's own environment, purpose in life, and the feeling of continuous personal growth. Swarbrick (2006) considers well‐being a holistic construct, including multiple areas of health and functioning, such as physical and spiritual health as well as possessing an integrated personality. Diener (2000; Diener, et al., 2009) defined well‐being as a general experience of pleasure, satisfaction with life, absence of negative affect, and presence of positive affect. Seligman (2011) developed the PERMA model to explain how the pursuit of well‐being can be achieved through a combination of positive emotions, engagement, relationships, meaning, and accomplishment. Keyes (2013) proposed the construct of social well‐being, as a positive state of affairs that individuals can derive from adaptation to society in terms of social integration, social acceptance, social contribution, and social coherence.

Most of the above consider well‐being from a strictly individual and psychological point of view. Excluding some noticeable exceptions such as Keyes' model of social well‐being (2013), the other models neglect to consider how people's well‐being extends to socio‐cultural domains (Petrillo et al., 2015). Recently, some scholars have argued for the need to expand our understanding of the good life towards a multidimensional vision of subjective, psychological, psychosocial, and socio‐community elements of well‐being (Sarriera & Bedin, 2017). As shown by Linton et al. (2016), several tools have been proposed to measure multidimensional well‐being. Fugl‐Meyer et al. (1991), for example, developed the Life Satisfaction Questionnaire‐9, which considers mental, physical, social well‐being, activities, and personal circumstances. The Personal Well‐being Index‐Adult (International Well‐being Group, 2013) also evaluates several domains of well‐being including the often‐neglected dimension of spiritual well‐being. Last, the Multicultural Quality of Life Index (Mezzich et al., 2011) and the Self‐Evaluated Quality of Life Questionnaire (Ventegodt et al., 2003) cover both global well‐being and all the domains considered by the previous tools.

Within this context, Prilleltensky and colleagues (2012, 2015) have proposed a multidimensional model of well‐being, which considers relevant life domains. This vision derives from the notions that well‐being is achieved by the simultaneous fulfilment of needs at the individual, relational, organizational, and community levels (Prilleltensky, 2012). The I COPPE model (Prilleltensky et al., 2015) considers well‐being across six domains: interpersonal, community, occupational, physical, psychological, and economic. To these, it also includes an overall assessment of one's life, namely overall well‐being. Interpersonal well‐being refers to the degree of satisfaction with one's intimate relationship with family, friends and colleagues; community well‐being refers to satisfaction with the place where we live; occupational well‐being refers to the level of satisfaction with one's main activity, such as work or caring for home and family; physical well‐being refers to one's general state of health; psychological well‐being refers to the degree of satisfaction with one's emotional life; and lastly, economic well‐being refers to one's financial condition (Prilleltensky et al., 2015).

To operationalise their theory of multidimensional well‐being, Prilleltensky and colleagues (2015) have developed the I COPPE scale. The I COPPE scale is a self‐administered tool that measures the above‐mentioned seven domains of well‐being through 21 items. This tool also has the important advantage of considering well‐being from a temporal perspective. In fact, each dimension of well‐being is measured by three items: present, past, and future well‐being. This aspect allows for a more stable evaluation of people's perception of well‐being over time. To date, the I COPPE scale has been adapted in several countries and across diverse social groups. In Italy, Di Martino et al. (2018) conducted an adaptation study with a national sample, which confirmed its psychometric validity. Lietz et al. (2018) used a shorter version of the scale (without the economic dimension) to assess university students' well‐being in Italy and Serbia. Myers et al. (2016) tested its validity with a sample of Hispanic people living in the United States. Based on the results obtained by Myers et al. (2014) and from the Italian adaptation of the I COPPE scale, which showed a lower relationship among items of the past and their corresponding domains of well‐being (Di Martino et al., 2018), we decided to develop a short form of the I COPPE scale that includes only present and future items.

This decision was also endorsed by theoretical and empirical evidence from scientific literature, which suggests that when it comes to people's self‐evaluation of well‐being, recollection of past events is more prone to errors (e.g., recollections bias, fading effect bias) than the assessment of present and future circumstances (Kahneman & Riis, 2005; Walker et al., 2003). Other studies also suggest that people's evaluation of past well‐being is less reliable since it changes based on people's either positive or negative attitude towards past events (Adler & Pansky, 2020; O'Brien et al., 2012).

Other validation studies also support this view. In fact, the Gallup (2009) concluded than only items of present and future ratings offered the best reliability for measuring well‐being. Pavot et al. (1998) reached similar conclusions in evaluating the Temporal Satisfaction with Life Scale. As the authors concluded: *“…the addition of the past items…. did not result in a significant increment in the prediction of peer‐reported SWB*” (p. 349).

## 2 METHOD

### 2.1 Participants and procedure

The findings presented in this article are derived from two studies. The purpose of the pilot study was to reduce the full version of the I COPPE scale from 21 items to 14, by excluding 7 items dealing with past well‐being. The main purpose of the pilot study was to derive some initial results from deleting the past component of well‐being from the I COPPE scale, before launching a full validation study.

Due to the exploratory nature of the pilot study, we decided to avoid costly primary data collection in this phase and relied instead on secondary data. This is not an unusual practice, which is sometimes recommended to validate the psychometric validity and reliability of short‐form instruments (Widaman et al., 2011).

The pilot study draws on data collected from three countries (i.e., United States, Italy, and Argentina), which were chosen for their diversity in terms of language, history, culture, and geography. The US sample—which was used to validate the original version of the scale—was collected in 2015 and is composed of 426 US citizens (212 males and 214 females) with a mean age of 50.86 years (*SD* = 13.57). For the demographic characteristics of this sample, we refer the readers to Prilleltensky and colleagues (2015).

The Italian sample was collected between April and July 2015 and composed of 2017 Italian people (807 males and 1210 females), with a mean age of 30.528 years (*SD* = 11.759). The demographic characteristics of this sample are available in the Italian adaptation of the I COPPE scale (see Di Martino et al., 2018).

The Argentinian sample was collected between September and November 2018. The sample was composed of 482 (175 males and 307 females) Argentines with a mean age of about 22 years (*SD* = 3.78). Unlike the Unites States and Italy, the adapted I COPPE scale to Argentina has not yet been validated and the data were collected on a predominantly local sample of university students living in the province of Tucuman. The participants' demographic characteristics are available in Table 1.

**Table 1:** Demographic characteristics of Argentinian sample and of Italian sample (short version)

	Argentinian sample (I COPPE reduced pilot version) *n* = 482 (pilot study)	Italian sample (I COPPE short form) *n* = 2682 (full validation study)
Variable	*M* = 22.45 (*SD* = 3.78)	*M* = 29.73 (*SD* = 12.79)
Age	*N* (%)	*N* (%)
*Sex*		
Male	175 (36.3%)	1034 (38.6%)
Female	307 (63.7%)	1648 (61.4%)
Marital status		
Single	435 (90.2%)	1092 (40.7%)
With partner	23 (4.8%)	981 (36.6%)
Married	18 (3.7%)	488 (18.2%)
Separated/divorced	1 (0.2%)	87 (3.2%)
Widower	0 (0.0%)	13 (0.5%)
Other marital status	1 (0.2%)	21 (0.8%)
Educational level		
Primary/middle school	N.A.	202 (7.5%)
High school	N.A.	1541 (57.5%)
Univ. degree	N.A.	770 (28.7%)
Postgraduate degree	N.A.	169 (6.3%)
Other educational level	N.A.	0 (0.0%)
Employment status		
Unemployed	N.A.	266 (9.9%)
Employed	N.A.	681 (25.4%)
Student	N.A.	1236 (46.1%)
Retired	N.A.	46 (1.7%)
Undeclared worker	N.A.	89 (3.3%)
Other employment status	N.A.	291 (10.9%)
Missing answers	N.A.	73 (2.7%)

Prompted by the positive results obtained from the pilot study, which is described in detail below, we launched a final validation study. The final validation study is to be considered the primary investigation for the validation of the I COPPE scale short form. The study relies on primary data collected from a new sample of participants who were presented with a short form of the I COPPE scale. In this case, the I COPPE short form does not include items dealing with past well‐being. The sample was collected between May and September 2019 and included 2682 Italian people (1034 females and 1684 males), with a mean age of 29.73 years (*SD* = 12.79). The demographic characteristics of this sample are shown in Table 1.

As can be seen from the data reported in Table 1 and in previous articles (see Di Martino et al., 2018; Prilleltensky et al., 2015), the four samples, despite being different in age and marital status, are quite homogeneous in gender. With the exception of the American sample in which the two categories have almost the same percentage (50%), in the other three samples the proportion of women is around 60% of the total number of participants. Data collection techniques employed to build our samples also varied. An on‐line survey panel company contacted the American participants through email, asking them to complete an online survey (see Prilleltensky et al., 2015). Compared to the United States, the studies conducted in Italy and Argentina required translating and back translating the I COPPE scale to ensure meaningful language equivalence between culturally diverse contexts (Brislin, 1970). In addition, the Italian and Argentinian studies employed a combination of random sampling and snowball sampling technique (see Di Martino et al., 2018).

Participants were recruited both from the research team's contacts network and through the contacts network of a group of undergraduate university students previously involved in the research. Students were invited to find potential participants amongst their contacts network, which, in turn, was asked to send the link to the questionnaire to other participants. For this purpose, the students were trained in Computer Assisted Telephone Interview methodology and overseen by senior researchers throughout the process of data collection. All participants were invited to fill in the questionnaire via the SurveyMonkey online platform, where research information and instructions on how to fill in the questionnaire were available. A Facebook group was also created to facilitate the dissemination of research materials amongst the data collectors as well as to better share the link to the online questionnaire.

### 2.2 Measures

As already noted, this study employed two versions of the I COPPE scale. In its original full version, the I COPPE scale asks participants to assess their level of well‐being across 7 domains, using a Cantril scale ranging from 0 (minimum) to 10 (maximum) (Prilleltensky at al., 2015). Each domain taps into an item that evaluates well‐being in the current moment (present), in the previous year (past), and next year (future). In the pilot study, participants from the three samples (i.e., United States, Italian, and Argentinian) were instructed to complete the full versions of the I COPPE scale, which includes 7 items of past well‐being, 7 items of present well‐being, and 7 items of future well‐being. During data analysis, we omitted the items of past well‐being. Henceforth we will refer to this abbreviated scale as the “I COPPE reduced pilot version”. In the final validation study conducted in Italy, participants completed a shorter form of the I COPPE scale, with only 14 items, 7 of present well‐being, and 7 of future well‐being. In this case the items concerning the past were never presented to participants. From now on we will refer to this version as “I COPPE short form”.

## 3 RESULTS

### 3.1 Data analysis

Data were analysed by means of MPLUS v.8 to calculate descriptive statistics and to implement the confirmatory factor analysis (CFA) models. Maximum likelihood robust was chosen as main estimator, given the presence of univariate and multivariate deviation from normality. To assess model fit, we relied on conventional cut‐off points (Hu & Bentler, 1999), which recommend: *χ* ^2^ test to be less than 0.05; Tucker–Lewis index and comparative fit index (CFI) more than 0.095; root mean square error of approximation (RMSEA) less than 0.05, and standardized root mean square residual  less than 0.08.

Moreover, given the well‐known sensitivity of *χ* ^2^ test to large sample size (Bentler & Bonett, 1980; Fornell & Larcker, 1981), we ignored its results. As an alternative, we included two more indices, namely Gamma hat (Fan & Sivo, 2007; 2009) and McDonald's non‐centrality index (McDonald, 1989). This choice was driven by the evidence that they have proven to be robust, amongst other things, to large sample size. The literature suggests as cut‐off values, Gamma hat more than 0.95 and (Mc) more than 0.90 (Hu & Bentler, 1999).

Missing values were treated with list‐wise deletion, causing a relatively small loss of cases in nearly all instances. Only in the full validation study, the Italian sample presents 261 missing cases, which amounts to 12.9% of the total sample. However, this does not pose any significant threat to the power of our main analyses. In fact, power analyses based on the RMSEA test of close fit (MacCallum et al., 1996) shows that a minimum sample of about 278 respondents is sufficient to reach a recommended power of .8. Since all cases examined exceed this minimum requirement, we can confident that our results did not incur into a Type II error.

### 3.2 Finding*s*

Following the structure of the original I COPPE scale (Prilleltensky et al., 2015), we first applied a 7‐factor correlated‐trait model to both the I COPPE reduced pilot version (pilot study) and to the I COPPE short form (full validation study). As we can see in Table 2, we found highly acceptable indices of model fit in each sample considered. Therefore, we can accept the null hypothesis that in each sample the model's implied variance‐covariance matrix [Σ(θ)] and the model's covariance matrix [Σ] are not significantly different. Amongst the samples that used the reduced pilot version of the I COPPE scale, the Argentinian sample presents the best fit to the data, yet with no substantial difference in model fit values compared to the Italian and American sample.

**Table 2:** Indices of model fit in the United States, Italian, Argentinian sample, and I COPPE short form

Instrument	Sample	*χ* ^2^ *_df_* (*p* value)	CFI/TLI	RMSEA (90% CI)	SRMR	Gamma hat	McDonald's non‐centrality index (Mc)
I COPPE reduced pilot version (pilot study)	US (*n* = 426)	47.042_35_ (.084)	0.997/0.991	0.028 (0.00, 0.048)	0.015	0.996	0.986
	Italian (*n* = 1978)	95.132_35_ (<.001)	0.993/0.983	0.029 (0.022, 0.037)	0.033	0.998	0.985
	Argentinian (*n* = 472)	25.790_35_ (.871)	1.00/1.01	0.000 (0.00, 0.017)	0.018	1.00	1.00
I COPPE short form (*n* = 2943) (full validation study)		83.755_35_ (<.001)	0.997/0.992	0.022 (0.016, 0.028)	0.016	0.997	0.991

Abbreviations: CFI, comparative fit index; CI, confidence interval; RMSEA, root mean square error of approximation; TLI, Tucker–Lewis index.

In the same vein, the I COPPE scale short form presents similar indices of model fit to the other samples. In terms of parameters estimates, Table 3 shows that both the I COPPE reduced pilot version and the I COPPE short form present highly significant factor loadings and inter‐item reliability values, with a minimum of 0.58 (*R* ^2^ = .34) for overall future well‐being in the Italian sample, and a maximum of 0.99 (*R* ^2^ = 0.98) for occupational present well‐being in the US sample.

**Table 3:** Factor loadings and inter‐item reliability (*R* ^2^) in the United States, Italian, Argentinian sample and I COPPE short form

		I COPPE reduced pilot version (pilot study)			I COPPE short form (full validation study)
Latent variable	Item	US Sample	Italian Sample	Argentinian sample	
		Standardized factor loadings (*R* ^2^)	Standardized factor loadings (*R* ^2^)	Standardized factor loadings (*R* ^2^)	Standardized factor loadings (*R* ^2^)
Overall well‐being	Overall well‐being (present)	0.96 (0.93)	0.92 (0.86)	0.91 (0.84)	0.96 (0.93)
	Overall well‐being (future)	0.82 (0.68)	0.58 (0.34)	0.68 (0.47)	0.73 (0.53)
Interpersonal well‐being	Interpersonal well‐being (present)	0.95 (0.90)	0.90 (0.81)	0.87 (0.77)	0.94 (0.89)
	Interpersonal well‐being (future)	0.88 (0.78)	0.78 (0.62)	0.82 (0.68)	0.73 (0.54)
Community well‐being	Community well‐being (present)	0.95 (0.91)	0.96 (0.92)	0.95 (0.91)	0.95 (0.90)
	Community well‐being (future)	0.91 (0.84)	0.75 (0.57)	0.82 (0.67)	0.85 (0.73)
Occupational well‐being	Occupational well‐being (present)	0.99 (0.98)	0.90 (0.81)	0.86 (0.74)	0.88 (0.78)
	Occupational well‐being (future)	0.86 (0.75)	0.64 (0.41)	0.77 (0.59)	0.73 (0.54)
Physical well‐being	Physical well‐being (present)	0.92 (0.86)	0.90 (0.82)	0.84 (0.70)	0.92 (0.86)
	Physical well‐being (future)	0.85 (.73)	0.70 (.49)	0.83 (.69)	0.75 (0.56)
Psychological well‐being	Psychological well‐being (present)	0.94 (.90)	0.93 (.86)	0.89 (.79)	0.93 (0.88)
	Psychological well‐being (future)	0.87 (.76)	0.63 (.40)	0.78 (.61)	0.73 (0.53)
Economic well‐being	Economic well‐being (present)	0.94 (.89)	0.90 (.81)	0.80 (.64)	0.87 (0.77)
	Economic well‐being (future)	0.81 (.66)	0.65 (.43)	0.70 (.49)	0.76 (0.58)
*N*		426	1978	472	2943

*Note*: N.B. all values are significant at 0.1% alpha level.

As reported in previous cases (Di Martino et al., 2018; Prilleltensky et al., 2015), the items of present well‐being show the highest loadings and inter‐item reliability.

### 3.3 Reliability and construct validity

The reliability of the 7 factors making up the I COPPE reduced pilot version and the I COPPE short form were assessed through composite reliability (CR), which has demonstrated to perform better than the most commonly used cronbach alpha, particularly in cases of non tau‐equivalence (Raykov, 1997). Values of CR higher than 0.7 are considered a sign of good reliability.

Convergent validity was assessed through average variance extracted (AVE), which has better performance properties than the most commonly used Campbell and Fiske's (1959) method, which relies on correlations between the target factors with other instruments deemed to measure similar constructs (Cheung & Wang, 2017). Values of AVE higher than 0.5 are indicative of good convergent validity. In addition, discriminant validity can be established if AVE is higher than both maximum squared shared variance (MSV) and average shared square variance (ASV) (Hair et al., 2010).

Table 4 shows that both the I COPPE reduced pilot version and the I COPPE short form present high values of Composite reliability, which range from a minimum of 0.723 for psychological well‐being in the Argentinian sample, to a maximum of 0.935 for interpersonal well‐being in the US sample. In terms of convergent validity, all the values of AVE exceed the threshold of 0.5, with a minimum value of 0.567 for psychological well‐being in the Argentina sample, and a maximum value of 0.877 for interpersonal well‐being in the US sample. In terms of discriminant validity, the values of AVE are always higher than both Maximum Shared Variance and Average Shared Variance. The only exception we found is for economic well‐being in the I COPPE short form. However, only 0.002 points of difference between AVE and MSV should not pose a serious threat to the discriminant validity of this factor.

**Table 4:** Factor correlations, reliability, and validity measures of the I COPPE scale reduced pilot version across US, Italy, and Argentina and I COPPE short from

Sample	Latent variable	IN_WB	CO_WB	OC_WB	PH_WB	PS_WB	EC_WB	OV_WB
US (*n* = 426) (pilot study)	IN_WB	1						
	CO_WB	0.545	1					
	OC_WB	0.493	0.526	1				
	PH_WB	0.424	0.499	0.579	1			
	PS_WB	0.576	0.614	0.682	0.714	1		
	EC_WB	0.487	0.617	0.657	0.591	0.677	1	
	OV_WB	0.604	0.62	0.723	0.754	0.8	0.75	1
	Reliability and validity measures							
	CR ($\rho_{c}$)	0.916	0.935	0.931	0.887	0.909	0.876	0.895
	AVE ($\rho_{\overset{®}{v}}$)	0.846	0.877	0.871	0.797	0.834	0.781	0.81
	MSV	0.365	0.384	0.523	0.569	0.640	0.563	0.640
	ASV	0.276	0.327	0.379	0.365	0.464	0.403	0.507
	Latent variable	IN_WB	CO_WB	OC_WB	PH_WB	PS_WB	EC_WB	OV_WB
Italian (*n* = 1978) (pilot study)	IN_WB	1						
	CO_WB	0.377	1					
	OC_WB	0.382	0.404	1				
	PH_WB	0.401	0.32	0.416	1			
	PS_WB	0.55	0.381	0.503	0.619	1		
	EC_WB	0.311	0.384	0.512	0.462	0.483	1	
	OV_WB	0.615	0.419	0.561	0.595	0.801	0.543	1
	Reliability and validity measures							
	CR ($\rho_{c}$)	0.833	0.901	0.797	0.832	0.828	0.807	0.844
	AVE ($\rho_{\overset{®}{v}}$)	0.717	0.82	0.664	0.714	0.71	0.678	0.734
	MSV	0.378	0.176	0.315	0.383	0.642	0.295	0.642
	ASV	0.204	0.146	0.219	0.231	0.326	0.208	0.360
	Latent Variable	**IN_WB**	**CO_WB**	**OC_WB**	**PH_WB**	**PS_WB**	**EC_WB**	**OV_WB**
Argentinian (*n* = 472) (pilot study)	IN_WB	1						
	CO_WB	0.282	1					
	OC_WB	0.379	0.197	1				
	PH_WB	0.356	0.309	0.448	1			
	PS_WB	0.476	0.328	0.519	0.641	1		
	EC_WB	0.348	0.367	0.452	0.411	0.472	1	
	OV_WB	0.553	0.215	0.643	0.505	0.672	0.377	1
	Reliability and validity measures							
	CR ($\rho_{c}$)	0.843	0.887	0.802	0.825	0.826	0.723	0.79
	AVE ($\rho_{\overset{®}{v}}$)	0.729	0.79	0.67	0.702	0.705	0.567	0.658
	MSV	0.306	0.135	0.413	0.255	0.452	0.223	0.452
	ASV	0.167	0.084	0.212	0.210	0.281	0.166	0.269
I COPPE short form (*n* = 2943) (full validation study)	Latent Variable	IN_WB	CO_WB	OC_WB	PH_WB	PS_WB	EC_WB	OV_WB
	IN_WB	1						
	CO_WB	0.286	1					
	OC_WB	0.290	0.385	1				
	PH_WB	0.398	0.305	0.358	1			
	PS_WB	0.530	0.378	0.534	0.523	1		
	EC_WB	0.255	0.336	0.553	0.396	0.41	1	
	OV_WB	0.527	0.422	0.608	0.474	0.778	0.467	1
	Reliability and validity measures							
	CR ($\rho_{c}$)	0.836	0.855	0.756	0.791	0.77	0.762	0.743
	AVE ($\rho_{\overset{®}{v}}$)	0.719	0.749	0.614	0.658	0.635	0.656	0.603
	MSV	0.281	0.178	0.370	0.274	0.605	0.306	0.605
	ASV	0.158	0.126	0.220	0.172	0.293	0.171	0.312

*Note*: N.B. All values are significant at the 0.1% alpha level.

Abbreviations: ASV, average shared square variance; AVE, average variance extracted, CR, composite reliability; MSV, maximum squared shared variance.

These results suggest that both the I COPPE scale reduced pilot version and the I COPPE short form present acceptable levels of reliability, convergent, and discriminant validity.

### 3.4 Model comparisons

Having established the psychometric proprieties of both the I COPPE scale reduced pilot version and the I COPPE short form, in this last paragraph we will report the results of comparisons of the I COPPE short form against alternative CFA structures. Although the I COPPE scale was previously tested against competing models (see Di Martino et al., 2018), this article set out to derive the I COPPE short form, which statistically speaking represents a somewhat new scale and therefore it ought to be tested against alternative model structures. On that account the I COPPE short form was tested against a second order solution (Model B), which constrains the 7 factors solution (Model A) to an additional latent variable explaining the 7 factors of well‐being; a Multi‐Trait Multi‐Method solution (Model C) where the 7 factors solution (Model A) is additionally constrained to two latent variables, one explaining all items of present well‐being and one explaining all items of future well‐being. Last, we compared a one factor solution (Model D), which builds on one latent variable explaining all 14 items of well‐being. A Bi‐factor solution was not included on this occasion because it was not possible to reach an identified solution. This condition is likely due to the complexity of the model.

When comparing nested models with large samples, the *χ* ^2^ difference test tends to suffer from the same shortcomings of absolute *χ* ^2^ test (Brannick, 1995). In our case, given 2943 observations, it is advisable not to trust results from *χ* ^2^ difference test. As an alternative, we followed Fan and Sivo's (2009) recommendations to compare difference in CFI, Gamma hat, and McDonald's non‐centrality index (NCI), given their robustness to large sample size. When comparing nested models, Cheung and Rensvold (2002) recommend a difference in value of ≤0.005 or 0.010 for CFI, ≤0.005 or 0.008 for Gamma hat, and ≤0.010 or 0.015 for McDonald's non‐centrality index.

Since the Multi‐Trait Multi‐Method (Model C) and the One factor solution (Model D) are nonnested compared to Model A, we will rely on differences in Bayesian information criterion (BIC) values, which are more robust to large sample size than Akaike information criterion (AIC). Kass and Raftery (1995), suggest the following guidelines to assess differences in BIC: between 1 and 3 = not worth mentioning, between 3 and 20 = positive, between 20 and 150 = strong, higher than 150 = very strong.

Results reported in Table 5 show that, consistent with the I COPPE scale full version, the 7‐factor correlated‐traits solution (Model A) presents better fit to the data than its competing models.

**Table 5:** Model comparisons between the I COPPE short from 7‐factor correlated‐traits model and alternative models

Model/indices	A 7 Factors correlated‐traits	B 2nd order	C Multi‐Trait Multi‐Method	D one factor
MLR *χ* ^2^	83.755	306.642	154.803	223.395
*χ* ^2^ *df*	35	49	41	39
*χ* ^2^ *p*	<0.001	<.001	<0.001	<0.001
CFI	0.997	0.984	0.993	0.988
TLI	0.992	0.970	0.984	0.973
RMSEA (90% CI)	0.022 (.016, .028)	0.042 (0.038, 0.047)	0.031 (0.026, 0.036)	0.040 (0.035, 0.045)
SRMR	0.016	0.034	0.015	0.029
Gamma hat	0.997	0.987	0.994	0.991
McDonald's non‐centrality index (NCI)	0.991	0.957	0.980	0.969
Akaike (AIC)	152940.135	153236.071	153018.804	153124.446
Bayesian (BIC)	153443.059	153655.174	153485.805	153603.421
Model comparison		B versus A	C versus A	E versus A
ΔCFI	/	−0.013	/	/
ΔGamma hat	/	−0.01	/	/
ΔNCI	/	−0.03	/	/
ΔBIC		212.115	42.746	160.362

With respect to the 2nd order solution (Model B), the difference in CFI, Gamma hat, and McDonald's NCI highly exceed the recommended thresholds thereby favouring the former solution. The values of BIC also indicate a strong difference between Model A and Model C, ΔBIC = 42.74, and a very strong difference between Model D and Model A, ΔBIC = 160.36, in both cases favouring the 7‐factor correlated‐traits solution.

**Figure 1:**
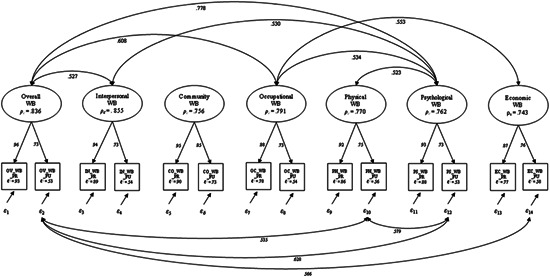
7‐Factor correlated‐trait model of I COPPE short form. N.B. All values are significant at the .1% alpha levelN.B.2 Only correlations more than 0.5 were depicted to reduce clutter* ρc = composite reliability; ℓ2 = inter‐item reliability; ε = measurement residuals

Figure 1 shows in a graphical format the I COPPE short form 7‐factor correlated‐trait model, along with factor loadings and inter‐item reliability values, and correlations between factors and between measurement residuals. As we can see in the figure, only correlations more than 0.5 are displayed to avoid cluttering. For a full list of factors correlations, we refer the readers to Table 4.

## 4 DISCUSSION

Our results lend support to the hypothesis that a shorter version of the I COPPE scale presents good psychometric proprieties across all samples considered. First, we carried out a pilot study to test whether the I COPPE scale could be restructured into a reduced pilot version by omitting items of past well‐being in the data analysis phase. Then we launched a final validation study to test responses to the I COPPE short form, which does not include items of past well‐being, even in the survey. In all cases examined, we found high levels of Composite Reliability and AVE, the latter exceeding, in all but one case, the values of Maximum Shared Variance and ASV. These results suggest that a shorter version of the I COPPE scale possesses good levels of both reliability as well as convergent and discriminant validity.

In terms of parameter estimates, all congeneric variables show high factor loadings and inter‐item reliability values with respect to their corresponding factors. The items of present well‐being show the highest values, indicating that people are more consistent and less biased in their present evaluations when trying to assess their state of well‐being.

Lastly, the I COPPE scale short form was tested against 3 competing models, namely a 2nd order solution, a Multi‐Trait Multi‐Method solution, and a one factor solution. Results from comparing differences in model fit indices for nested models and BIC values for nonnested models revealed that, consistent with the I COPPE scale full version, its short form is best expressed through a model with 7 factors correlated‐traits, with each factor (or domain of well‐being) tapping into one item of past well‐being and 1 item of future well‐being.

## 5 LIMITATIONS AND RECOMMENDATIONS

An important limitation to consider when analysing data collected through the I COPPE scale short form is that each of the 7 latent variables that form the domains of well‐being are explained by only two manifest variables. As we are aware, the literature encourages the use of more than two parameters in confirmatory factor analysis, for reasons of identification (Bollen & Davis, 2009). In fact, a limited number of parameters per factor is likely to generate improper solutions. The most common we encountered for the I COPPE short form is the presence of Heywood cases with negative measurement error variances (Bentler & Chou, 1988). This is particularly likely to happen when data are collected from small samples (Gerbing & Anderson, 1987).

Several solutions have been proposed to deal with Haywood cases (see Chen et al., 2001; Dillon et al., 1987). In our case, we managed to avoid altogether incurring into Haywood cases by correlating the residual errors of all the items of future well‐being, rather than the ones of present well‐being. However, from a statistical point of view, we still recommend the use of the full version of the I COPPE scale whenever possible to avoid Haywood cases altogether. Whenever a justifiable choice is made for using its short form, we recommend collecting relatively large samples, carefully inspecting the residual matrix, and also adopting the strategies described by Chen and colleagues (2001).

Although the main validation of the I COPPE short form gave positive results, we recommend caution in generalising the findings beyond the Italian context. This is appearing even more relevant in view of some lack of homogeneity amongst the samples collected. This is especially true for the US and the Argentinian samples, where the difference between the mean age of the participants is approximately 28 years. Therefore, it is desirable that future research test the validity of the I COPPE scale short form with samples as homogeneous as possible.

One last limitation refers to the amount of missing data in the final validation study. Although we demonstrated that the reduction of nearly 13% of the sample due to the deletion of missing data, does not harm the statistical power of our main tests, we should still be mindful that this could influence the generalizability of our results.

## 6 CONCLUSIONS

The study confirms that the I COPPE scale short form, which includes only present and future well‐being items, presents high validity and reliability in the measurement of well‐being. This result is in line with previous analysed conducted by Myers et al. (2014) on the I COPPE scale, which concluded that “…*an individual's perceptions of the past, at least in some circumstances, may offer negligible empirical contributions over and above an individual's perceptions of the present and future in the practical assessment of multidimensional well‐being*” (p. 796).

Moreover, our findings suggest that the I COPPE scale short form could be used in different countries, given that we found similar psychometric properties across the United States, Italian, and Argentinian samples. However, we should also be mindful that the people from the pilot study, who completed the I COPPE scale reduced pilot version were presented with a somewhat different version of the final I COPPE short form, which was validated in the final validation study. Future studies might bring further evidence to the validity of the I COPPE short form in other countries other than Italy. The universality of the I COPPE scale short form would be better assessed through more suitable statistical techniques such as multigroup invariance as well as a higher number and more diverse type of countries.

Last, we should acknowledge some of the advantages that a short form of the I COPPE scale offers to those interested in measuring multidimensional well‐being. First of all, a shorter number of items allows for less time to complete the survey, with a consequent reduction of biases due to respondent fatigue (Ben‐Nun, 2008). Furthermore, sometimes using too many items in the same survey can be counterproductive, in that the multidimensionality of a construct gets confused with the multiplicity of factors that cause it (Bowling, 2005). Instead, fewer carefully selected items help reduce the complexity of an instrument without undermining its multidimensionality, which is fundamental to acknowledge the complex nature of well‐being.

In conclusion, professionals interested in the measurement of well‐being can benefit from using the I COPPE short form whilst still being confident enough in the validity and reliability of their results.

7

### PEER REVIEW

The peer review history for this article is available at https://publons.com/publon/10.1002/jcop.22659

## CONFLICT OF INTERESTS

The authors declare that there are no conflict of interests.

## ETHICS STATEMENT

The study protocol was approved by the ethics committee of the University of Naples Federico II.

## Data Availability

Data available on request from the authors.
